# Altered expression of glycan patterns and glycan-related genes in the medial prefrontal cortex of the valproic acid rat model of autism

**DOI:** 10.3389/fncel.2022.1057857

**Published:** 2022-12-08

**Authors:** Yingxun Liu, Yuanyuan Di, Qi Zheng, Zhaoqiang Qian, Juan Fan, Wei Ren, Zhaoming Wei, Yingfang Tian

**Affiliations:** ^1^Key Laboratory of Ministry of Education for Medicinal Plant Resource and Natural Pharmaceutical Chemistry, National Engineering Laboratory for Resource Developing of Endangered Chinese Crude Drugs in Northwest of China, College of Life Sciences, Shaanxi Normal University, Xi’an, Shaanxi, China; ^2^College of Life Sciences, Shaanxi Normal University, Xi’an, Shaanxi, China; ^3^Genetic Engineering Laboratory, College of Biological and Environmental Engineering, Xi’an University, Xi’an, Shaanxi, China; ^4^School of Education, Shaanxi Normal University, Xi’an, Shaanxi, China

**Keywords:** glycan-related genes, glycan patterns, valproic acid, autism, mPFC

## Abstract

Autism spectrum disorders (ASD) represent a group of neurodevelopmental defects characterized by social deficits and repetitive behaviors. Alteration in Glycosylation patterns could influence the nervous system development and contribute to the molecular mechanism of ASD. Interaction of environmental factors with susceptible genes may affect expressions of glycosylation-related genes and thus result in abnormal glycosylation patterns. Here, we used an environmental factor-induced model of autism by a single intraperitoneal injection of 400 mg/kg valproic acid (VPA) to female rats at day 12.5 post-conception. Following confirmation of reduced sociability and increased self-grooming behaviors in VPA-treated offspring, we analyzed the alterations in the expression profile of glycan patterns and glycan-related genes by lectin microarrays and RNA-seq, respectively. Lectin microarrays detected 14 significantly regulated lectins in VPA rats, with an up-regulation of high-mannose with antennary and down-regulation of Siaα2-3 Gal/GalNAc. Based on the KEGG and CAZy resources, we assembled a comprehensive list of 961 glycan-related genes to focus our analysis on specific genes. Of those, transcription results revealed that there were 107 differentially expressed glycan-related genes (DEGGs) after VPA treatment. Functional analysis of DEGGs encoding anabolic enzymes revealed that the process trimming to form core structure and glycan extension from core structure primarily changed, which is consistent with the changes in glycan patterns. In addition, the DEGGs encoding glycoconjugates were mainly related to extracellular matrix and axon guidance. This study provides insights into the underlying molecular mechanism of aberrant glycosylation after prenatal VPA exposure, which may serve as potential biomarkers for the autism diagnosis.

## Introduction

Autism spectrum disorder (ASD) are characterized by early-onset difficulties, including social deficits, and abnormally restricted and repetitive behaviors (American Psychiatric Association, 2013; Muhle et al., 2018). To date, the exact pathophysiology and molecular mechanism of autism still remain unclear, because of the complex interplay of environmental and genetic factors that determine susceptibility (Kuo and Liu, 2018; Muhle et al., 2018). Gene-environment interactions may influence the expression of genes that are mainly related to neuronal activity and synapse development during the early stage of brain development (Mabunga et al., 2015; Varghese et al., 2017). Thus, pathophysiological processes in ASD seem to converge on specific molecular networks and pathways, with a precise regulation in the formation and function of brain synapses (Quesnel-Vallieres et al., 2019).

Glycosylation is one of the most common and frequent post-translational protein modifications (Maupin et al., 2010). Glycans and their conjugates (glycoproteins, proteoglycans, and glycolipids) are the major constituents of the neural extracellular matrix (ECM) (Dwyer and Esko, 2016; Schjoldager et al., 2020). In this context, glycosylation can influence the nervous system in various ways by affecting the functions of glycoproteins involved in nervous system development and physiology (Mutalik and Gupton, 2021). The production of glycan and glycoconjugate is governed by a series of glycan biosynthesis and catabolic enzymes. In contrast, alterations in the expression of these critical enzymes sensitively reflect the glycosylation patterns, cell morphology, and pathogenesis of neurodevelopmental disorders, including ASD (Blanchard et al., 2012; Irie et al., 2012; Pearson et al., 2013; Freeze et al., 2015; Yang et al., 2018; Demirci et al., 2019; Oinam et al., 2020; Yang et al., 2020). Demirci et al. (2019) provided evidence that the levels of salivary sialic acid (Sia) in children with ASD were statistically lower than those in healthy controls. Furthermore, the expression of the *GNE* gene, which encodes UDP-GlcNAc2-epimerase/ManNAc kinase, a key enzyme in Sia biosynthesis, was reported to be negatively correlated with stereotypical behaviors in children with ASD (Yang et al., 2018, 2020). Heparan sulfate proteoglycan is one of the major components of the ECM, which is tightly connected to the intracellular environment, and required for cortical neurogenesis and axon guidance (Dwyer and Esko, 2016). Pagan et al. reported a reduction in the levels of heparan sulfate in young to mature autistic patients’ brain lateral ventricles (Pearson et al., 2013). These data agrees with previous findings from the BTBR T+tf/J mouse, a kind of inbred strain mice which displays an ASD-like behavioral phenotype (Blanchard et al., 2012), and with mice with genetic modifications reducing heparan sulfate (Irie et al., 2012). Thus, recent studies indicated that the aberrant protein glycosylation is potentially involved in the molecular mechanism of ASD patients and the genetic animal model of ASD.

**FIGURE 1:**
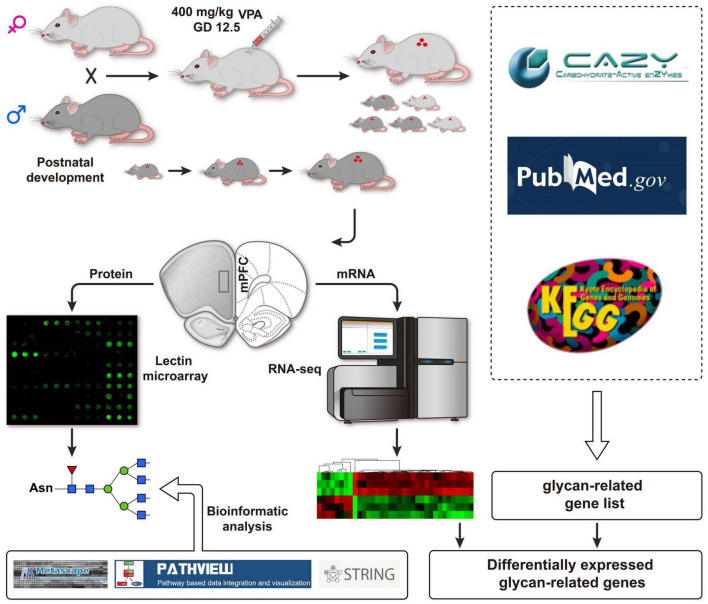
Schematic flow diagram of the integrated strategy used herein.

Embryonic exposure to valproic acid (VPA) in rodents is the most frequently used environmentally triggered autism model, which appropriately replicates disturbed behaviors, including social deficits and repetitive behaviors seen in ASD (Mabunga et al., 2015). VPA is non-specific histone deacetylase (HDAC) inhibitor and regulates gene transcription through chromatin remodeling (Fukuchi et al., 2009; Lucchina and Depino, 2014; Olde Loohuis et al., 2017; Zhang et al., 2018; Tartaglione et al., 2019). Therefore, we hypothesized that VPA treatment might cause the expression of glycan-related genes, which may contribute to protein glycosylation and affect perineuronal nets. As shown in Figure 1, we combined glycomics and transcriptomics analysis to systematically identify the aberrant expression of glycan patterns and glycan-related genes in a VPA-induced rat model of autism, which may provide useful insights into autism pathogenesis, and serve as potential biomarkers for autism diagnosis (Kuo and Liu, 2018).

## Materials and methods

### Valproic acid-induced rat model of autism

Sprague-Dawley rats were purchased from Xi’an Jiaotong University animal center (Xi’an, China). All rats were housed under a 12-h light/dark cycle at 18–22°C, relative humidity at 50–60%, and allowed free access to food and water. All animal experiments were performed under the National Institutional Animal Care and the guidelines approved by the Animal Care and Use Committee of Shaanxi Normal University (2019-021). All efforts were made to minimize the number of animals used and their suffering, and the spare rats were euthanized by carbon dioxide asphyxiation by using animal euthanasia device with the condition of 70% of the chamber or cage volume/min.

The VPA-induced rat model of autism was carried out as previously reported (Zhou et al., 2016; Zhang et al., 2018). Female rats (250–300 g) were mated overnight, the vaginal secretion was collected the next morning, and the day on which spermatozoa were detected was designated the first day of gestation (GD1). Pregnant female rats (*n* = 12) were randomly grouped into control (*n* = 5) and VPA groups (*n* = 7). The VPA group received a single intraperitoneal injection of 400 mg/kg VPA (250 mg/ml in saline, pH 7.3. Sigma, Oakville, CA) on GD 12.5, while the control group was injected with saline. The offspring were weaned on a postnatal day (PND) 23 and separated by sex. Only male offspring were used in the current study (Zhang et al., 2018). To minimize the litter-specific effects, only 1–2 animals per litter were used in the behavioral experiment, and one animal per litter was used in other analyses. All experiments were performed in a blinded manner in order to prevent subjective bias.

### Behavioral testing

To confirm the autist-like behaviors in rats prenatally exposed to VPA, the social behaviors and repetitive behaviors tests were performed from PND 30–35 on VPA (*n* = 11), and saline (*n* = 8) treated dams. The social behaviors test was performed using the three-chamber test as previously described (Wu et al., 2020; Di et al., 2021). Rats were individually acclimated for 5 min in the three-chamber apparatus 1 day before the test. At the beginning of the sociability test, the test rat was introduced into the center of the middle chamber for 5 min habituation. Then, an age- and sex-matched unfamiliar stimulus Sprague-Dawley rat (stranger) was placed in one of the side cages. The sociability test was performed for 10 min by placing the test rat into the middle chamber. Sociability was evaluated by the sociability index (SI), which was defined as the ratio of the duration of the test rat on the novel rat side to that on the empty side.

A self-grooming test was performed to estimate repetitive behaviors. Each rat was placed in a clean empty plastic cage (46 × 32 × 20 cm) and habituated for 10 min. The bedding was not used to prevent digging behavior. Then, the subject rat was recorded for cumulative time spent on self-grooming during the 10-min testing period. Grooming was defined as rubbing the face, body, or head with the two forelimbs.

### Medial prefrontal cortex collection

After the behavioral tests, animals were euthanized with 10 g/L with pentobarbital sodium (40 mg/kg) intraperitoneally. The mPFC region (AP = 3.7∼2.2 mm; ML = 0∼1.2 mm; DV = −2∼−5.0 mm) was dissected from both hemispheres according to the previous protocols with minor modifications (Völgyi et al., 2017; Sang et al., 2018). These tissues were rapidly frozen in liquid nitrogen and used for total protein and RNA extraction, respectively.

### Lectin microarrays and data analysis

Three male rats from VPA-exposed and control groups, were selected for lectin microarray. The mPFC tissue was harvested, and total proteins were extracted by lysing cells with PBST buffer (10 mM PBS (pH7.4), 140 mM NaCl, 2.7 mM KCl, 1% Triton X-100). The supernatant was collected after centrifugation at 14,000 × *g* for 20 min at 4°C. Following the protein concentration assay, equal amounts of proteins from different experiments were labeled with Cy5.5 fluorescence dye (GE Healthcare; Perry Hall, MD, USA) according to the manufacturer’s instructions. Briefly, Cy5.5 fluorescence dye was dissolved in DMSO for 0.5 h at room temperature, and it was incubated with proteins in 0.1 M Na_2_CO_3_ solution (pH 9.3) for 2.5 h at room temperature. The reaction was terminated by the addition of 4 M hydroxylamines for 10 min at 4°C. The Cy5.5 labeled proteins were purified by Sephadex G-25 columns, and applied to the lectin microarray.

The manufacture of lectin microarray and data acquisition was performed as described previously (Yu et al., 2012; Qin et al., 2017). Briefly, 4 μg of Cy5.5-labeled proteins were mixed with 0.5 mL of hybridization buffer and were incubated with the lectin microarray at 20°C overnight. Microarrays were washed with probing pad three times and centrifuged to dry. Finally, the microarrays were scanned using a GenePix 4000B confocal scanner. The GenePix Pro 3.0 software program extracted numerical data from the scanned images. The average background was subtracted, and values less than the average background ± 2 standard deviations (SD) were removed from each data point. The median of the effective data points for each lectin was globally normalized to the sum of the medians for all of the practical data points in one block. Each sample was consistently observed from three repeated slides. The normalized data of the VPA group and the control group were then compared to determine any relative change in protein glycosylation levels.

### Transcriptomic analysis of glycan-related genes expression

The expression profiles of glycan-related genes were analyzed by using RNA-seq with RNA isolated from the mPFC tissue of three VPA-exposed rats and control rats, respectively. The preparation of sequencing libraries, Illumina HiSeq2000 sequencing, and RNA-seq data processing were performed as described previously and following the manufacturer’s instructions (Zhang et al., 2018). Sample description has been deposited in the BioSample Submission Portal as Bioproject PRJNA397961, and complete data sets have been submitted to the Sequence Read Archive database^1^ under accession numbers SRR5950172 to SRR5950177. Gene expression in VPA-exposed rats was compared to saline-treated controls, and analysis was done using the DESeqR package. Genes with false discovery rate (FDR) adjusted *p*-value < 0.05 were assigned as differentially expressed genes (DEGs).

### Compilation of the glycan-related gene list

To focus our analysis on specific genes, we selectively analyzed the transcriptomics data from a target glycan-related genes list based on several sources, including Kyoto Encyclopedia of Genes and Genomes (KEGG^2^), Carbohydrate Active Enzymes (CAZy^3^), and National Center for Biotechnology Information (NCBI^4^) (see Table 1 for gene list categories, member totals, and sources). Unique NCBI gene identifiers (GeneIDs) for each member of the gene list were used to check for isoforms of a single gene to prevent duplications of gene entries in the list. Genes that encode anabolic enzymes with multiple functions were grouped by their catalytic activity to avoid redundant entries. Genes with adjusted *p*-value < 0.05 were assigned as differentially expressed glycan-related genes (DEGGs).

**TABLE 1:** Organization of the rat glycan-related gene list.

Group	Family or subgroup	No.	Source
**Enzymes**			
Glycosyltransferases	CAZy families: GT1-4, GT6-8, GT10-14, GT16-18, GT21-25, GT27, GT29, GT31-GT32, GT35, GT39, GT41, GT43, GT50, GT54, GT57-59, GT61, GT64-66, GT68, GT90, GT98, GT110, GT NC	259	CAZy
Glycoside hydrolases	CAZy families: GH1-2, GH13, GH18, GH20, GH22, GH27, GH29-31, GH33, GH35, GH37-38, GH47, GH56, GH59, GH63, GH65, GH79, GH84, GH99, GH116	94	CAZy
Carbohydrate-binding modules	CAZy families: CBM20-21, CBM48, CBM57	7	CAZy
Glycan biosynthesis and metabolism	N-Glycan biosynthesis	12	KEGG
	Various types of N-glycan biosynthesis	2	KEGG
	Mannose type O-glycan biosynthesis	5	KEGG
	Glycosaminoglycan biosynthesis	78	KEGG
	Glycosaminoglycan degradation	6	KEGG
	Glycosylphosphatidylinositol (GPI)-anchor biosynthesis	20	KEGG
	Glycosphingolipid biosynthesis–ganglio series	1	KEGG
	Other glycan degradation	1	KEGG
**Proteins**			
Lectins	C-Type, I-type, L-type, M-type, P-type, S-type, F-box lectins, Calnexin/calreticulin, Chitinase-like lectins, Intelectins	128	KEGG: Lectins
Glycosylphosphatidylinositol (GPI)-anchored proteins	Enzymes	18	KEGG
	Receptors	16	KEGG
	Antigens	67	KEGG
	Others	15	KEGG
Proteoglycans	Cell surface proteoglycans	12	KEGG
	ECM proteoglycans	36	KEGG
Glycosaminoglycan binding proteins	Heparan sulfate/Haparin	166	KEGG
	Hyaluronan	18	KEGG

### Verification by quantitative real-time PCR

Quantitative real-time PCR analysis was used to validate the glycan-related gene identified by RNA-seq. The sequence of gene-specific primers employed in the analysis is presented in Table 2. qRT-PCR was performed in CFX96™ Real-Time System (Bio-Rad, Hercules, CA, USA) as previously described (Zhang et al., 2018). Each PCR process was followed by a general dissociation curve protocol to check product specificity. All samples were run in biological triplicate, and the average values were calculated. The mRNA expression of the target gene was calculated by the △△CT method, and β-actin was employed as the reference gene, which is the main component of cytoskeletal protein with stable expression through various conditions.

**TABLE 2:** Primers and amplicon characteristics for the evaluated genes.

Gene symbol	Accession number	Primer sequences	Amplicon size/bp
*Large1*	NM_001108439.1	TCCTTGGCTGACCAGGACAT	200
		AGAGAGCCAGACTGATGGGT	
*Man2a2*	XM_008759529.3	TCTGTCTTAGCCTGCGCATC	116
		AGAGGGCGGAGGCGG	
*St6gal2*	XR_005488860.1	CTCCTGCAGCACAGCACTT	231
		TTGTTCAGACTCTGGCGGAC	
*Galnt9*	XM_039089515.1	CAACCAGCTGAATGAACGCT	122
		TCGTGGGTGGCTGTGATTTT	
*Hs3st5*	NM_001106392.1	GCCGAGCATCCCAAACTACT	127
		ACTCTGTCCTCACCGTTGTC	
*Actin*	NM_031144.3	TGACAGGATGCAGAAGGAGA	104
		TAGAGCCACCAATCCACACA	

### Analysis of biological processes of genes encoding anabolic enzymes

Functional analysis was performed by using Metascape^5^ tool to gain insights into functional groupings within the set of identified differentially expressed glycan-related genes. For the presentation of the expression profile data, fold change (FC) of the DEGGs encoding anabolic enzymes were uploaded to the Pathview web^6^ to gain color pathway visualization (Luo et al., 2017). The symbol nomenclature for glycans was used to visualize the glycan structures (Neelamegham et al., 2019).

### Protein interaction network analysis of genes encoding glycoconjugates

Protein interaction network analysis was performed as previously described (Cao et al., 2021). Briefly, protein interaction networks were extracted from the STRING database^7^ using a confidence cut-off of 0.4 (medium confidence level in STRING) for target genes encoding glycoconjugates. Markov cluster (MCL) was performed on the network in Cytoscape using Clustermaker2. For each functional neighbor gene cluster, we conducted gene ontology (GO) and KEGG pathway enrichment analysis using the Cytoscape plugin BiNGO based on universal GO, and KEGG annotation terms. The significant enriched biological processes (BP), molecular functions (MF), cellular component (CC), and KEGG pathway were highlighted with FDR value.

### Statistical analysis

GraphPad Prism 8 software (GraphPad Software, La Jolla, CA, USA) was used to generate descriptive statistics, perform statistical comparisons, and graph all continuous variables of behavioral testing. The normality test was performed by the Shapiro-Wilk test. Data sets that fit a normal distribution (*p* > 0.05) were compared using the standard unpaired *t*-test to determine significant differences between the two groups. When the *p*-value was less than 0.05, it was considered that the difference was statistically significant.

## Results

### Prenatal exposure to valproic acid results in autist-like behaviors

**FIGURE 2:**
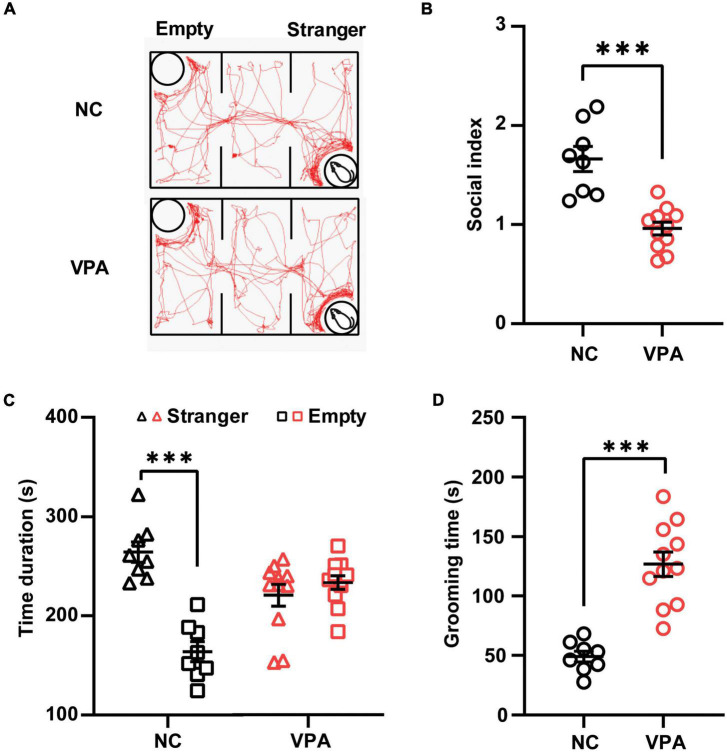
Effects of prenatal exposure to valproic acid (VPA) on autist-like behaviors in male offspring. **(A)** Track of rat offspring in the social test of the three-chamber test. Quantification of time duration **(B)** and the social index **(C)** in the social test showed decreased social behaviors of VPA-exposed offspring. **(D)** VPA-exposed offspring spent significantly more time on self-grooming during the 10-min test session. ^***^p ≤ 0.001.

Three-chamber test was employed to elucidate the social behavior deficits in the rat offspring prenatally exposed to VPA (Figure 2A). In the sociability stage of the test (Figure 2B), the control rats spent more time in the stranger chamber than those in the empty section [t(14) = 6.996, *P* < 0.0001], whereas the rats in the VPA group showed no significant difference in the time duration between the stranger and empty chamber [Stranger 220.5 ± 36.5, Empty 233.4 ± 23.4; t(20) = 0.9867, *P* = 0.3356]. Thus, the rats in the VPA group demonstrated a significant decrease in the social index compared with those in the control group {[t(17) = 5.374, *P* < 0.0001]; Figure 2C}. In self-grooming test (Figure 2D), VPA-treated rats showed a significant increase in total time spent self-grooming compared to the saline-injected controls [VPA 126.8 ± 36.5, Control 48.9 ± 13.0; t(17) = 6.104, *P* < 0.0001]. Taken together, rat offspring prenatally exposed to VPA at E12.5 exhibited autist-like behaviors, including social deficits and repetitive behaviors.

### Alteration of glycan patterns in the valproic acid-induced model of autism

**FIGURE 3:**
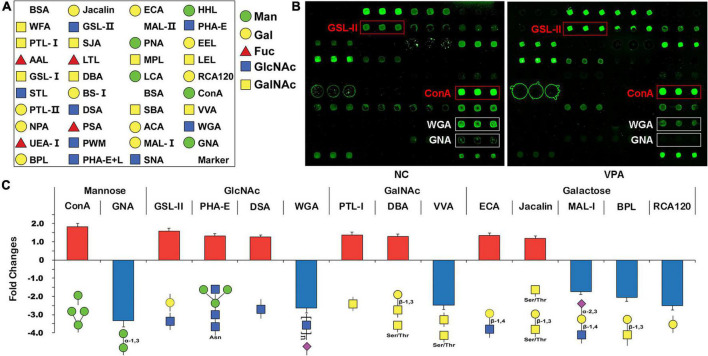
Glycan profiling of valproic acid (VPA) rats and negative control using lectin microarrays. **(A)** The layout of the lectin microarray. Each lectin is printed in triplicate. **(B)** Scanned images were derived by analysis of VPA rats and negative control. The substantially altered lectins [concanavalin A (ConA), *Griffonia simplicifolia* II (GSL-II), wheat germ agglutinin (WGA), and *Vicia villosa* agglutinin (VVA)] are marked with white boxes. **(C)** Fold change of normalized fluorescence intensity (NFI) of 14 lectins with significant differences in lectin microarray. NFI is the ratio of the median of the effective data points for each lectin to the sum of the medians for all of the practical data points for each lectin in one block. Lectins with fold change ≥ 1.2 or ≤ –1.2 were assigned as up-regulation or down-regulation.

To estimate the alteration of the glycosylation state after prenatal VPA exposure, the proteins were extracted from the mPFC of VPA-treated and saline-treated control rats and subjected to lectin microarrays. The layout of the lectin microarray, and the resulting glycopatterns defined by the microarrays for the VPA-treated and control groups are shown in Figures 3A,B. There were 14 lectins that significantly changed (FC ≥ 1.2 or ≤ −1.2) in VPA-treated rats compared to negative controls (Figure 3C; Table 3). Of those, ConA (FC = 1.82) and GNA (FC = −3.34) were the two most significantly up-regulated and down-regulated lectins, respectively. Although both bind to high mannose, the subtle mannose structure is different. ConA mainly binds branched mannose, while GNA specifically recognizes terminal α-1, 3 mannose, suggesting the modest mannose structure changed significantly in VPA-exposed rats. The remaining significantly changed lectins also included 4 GlcNAc-binding lectins (GSL-II, PHA-E, DSA, and WGA), 3 GalNAc-binding lectins (PTL-I, DBA, and VVA), 5 Galactose-binding lectins (ECA, Jacalin, MAL-I, BPL, and RCA120). Based on the specificity of lectin-recognized structures, the results of lectin microarrays revealed that complex-type N-glycan with antennary (recognized by GSL-II and PHA-E) was up-regulated. In contrast, the Siaα2-3 Gal/GalNAc (recognized by WGA and MAL-I) and terminal GalNAc structure (recognized by BPL and VVA) were down-regulated in VPA rats. In summary, lectin microarray results indicated that the glycan patterns of glycoproteins, especially the subtle high-mannose structure, were significantly altered in an environmentally triggered autism model.

**TABLE 3:** Changes in glycan structure recognized by 14 different lectins in valproic acid (VPA)-induced autism model.

Mono-saccharide	Lectin	Specificity	Binding structure	NFI		FC
				CON	VPA	
Mannose	ConA	Branched and terminal Man; terminal GlcNAc High-mannose, Manα1-6 (Manα1-3)Man	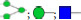	0.109	0.198	**1.82**
	GNA	High-mannose; Manα1-3Man	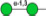	0.020	0.006	**−3.34**
GlcNAc	GSL-II	GlcNAc and agalactosylated tri/tetra antennary glycans	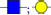	0.059	0.094	**1.59**
	PHA-E	Bisecting GlcNAc, biantennary complex-type N-glycan with outer Gal	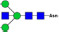	0.031	0.040	**1.32**
	DSA	β-D-GlcNAc, (GlcNAcβ1-4)n, Galβ1-4GlcNAc	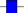	0.027	0.034	**1.26**
	WGA	Multivalent Sia and (GlcNAc)_n_	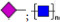	0.083	0.031	**−2.63**
GalNAc	PTL-I	GalNAc; GalNAcα-1,3Galβ-1,3/4Glc	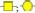	0.042	0.058	**1.38**
	DBA	GalNAcα-Ser/Thr(Tn); GalNAcα1-3((Fucα1-2))Gal	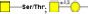	0.006	0.008	**1.29**
	VVA	terminal GalNAc; GalNAcα-Ser/Thr(Tn), GalNAcα1-3Gal	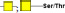	0.071	0.029	**−2.47**
Galactose	ECA	Galβ-1,4GlcNAc (type II); Galβ1-3GlcNAc (type I)	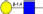	0.054	0.072	**1.34**
	Jacalin	Galβ1-3GalNAcα-Ser/Thr(T); GalNAcα-Ser/Thr(Tn)	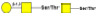	0.102	0.122	**1.20**
	MAL-I	Siaα2-3Galβ1-4Glc(NAc), Siaα2-3Gal	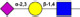	0.024	0.014	**−1.72**
	BPL	Galβ1-3GalNAc; Terminal GalNAc	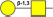	0.049	0.024	**−2.06**
	RCA120	β-Gal, Galβ-1,4GlcNAc (type II), Galβ1-3GlcNAc (type I)	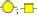	0.024	0.009	**−2.50**

NFI, Normalized fluorescent intensity; FC, Fold change.

### Assembly of the glycan-related gene list

To examine the global regulation of glycan abundance in rat brain tissue, we first generated a comprehensive list of glycan-related genes, which included genes encoding glycosyltransferases (GTs), glycoside hydrolases (GHs), anabolic enzymes related to glycosylation, and glycan-binding proteins. For assembly of the gene list, numerous web-based resources were chosen, predominantly CAZy database^3^ and KEGG glycan.^8^ We first added the 259, 94, and 7 rats genes encoding GTs, GHs, and carbohydrate-binding modules, respectively, that are registered in the CAZy database (Nairn et al., 2008). Subsequently, 125 genes encoding anabolic enzymes other than GTs or GHs were added, such as genes involved in N-Glycan and O-glycan biosynthesis, Glycosaminoglycan biosynthesis and degradation, Glycosylphosphatidylinositol (GPI)-anchor biosynthesis and Glycosphingolipid biosynthesis. Finally, 476 genes encoding glycan-binding proteins (lectins), Proteoglycans, GPI-anchored Proteins, or Glycosaminoglycan Binding Proteins were accumulated from the KEGG glycan database. In total, 961 rats genes were collected and categorized into eight groups. The complete list of rat glycan genes, including gene list categories, member totals, and sources, is contained in Table 1.

### Identification of differentially expressed glycan-related genes in valproic acid-induced rat model of autism

**FIGURE 4:**
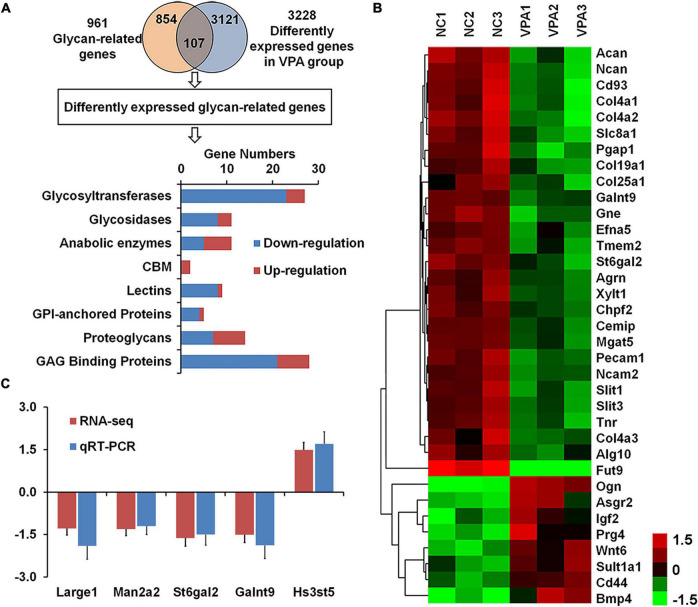
Identification of differentially expressed glycan-related genes in valproic acid (VPA)-induced autism model. **(A)** VennPlex representation of the glycan-related genes from our glycan-related genes list (yellow) and differentially expressed genes (DEGs) from VPA rats (blue). The histogram in the lower part shows the classification of 107 differentially expressed glycan-related genes according to the gene category. **(B)** The expression of 35 differentially expressed glycan-related genes with fold change of more than 1.5 fold are clustered and shown as a heatmap. All signals are compared to a mean value, and fold difference from the of mean is visually represented by color assignment (see the scale on the right side). Green dots represent the significantly down-regulated genes, and red dots represent the significantly up-regulated genes. **(C)** Gene expression of *Large1, Man2a2, St6gal2, Galnt9*, and *Hs3st5* was confirmed by quantitative real-time PCR.

To explore the causes of the alteration in glycan patterns, we used RNA sequencing to investigate transcriptome in the prefrontal cortex of VPA-exposed rats, and found 3,228 differently expressed genes in VPA rats compared to controls (adjusted *p*-value < 0.05) (Figure 4A; Zhang et al., 2018). To focus our analysis on specific genes, we generated a comprehensive rat glycan-related gene list based on the KEGG glycan and CAZy database (Kanehisa et al., 2016). Of these 961 glycan-related genes, the mRNA levels of 107 genes significantly changed in VPA-treated rats (Supplementary Table 1). As shown in Figure 4A, most DEGGs fall under glycosyltransferases (27 genes, 25.2%) and glycosaminoglycan binding proteins (28 genes, 26.1%). More information about these DEGGs was further categorized according to their specific functions and listed in Tables 4, 5.

**TABLE 4:** The expression changes of glycan genes encoding anabolic enzymes involved in glycosylation in valproic acid (VPA)-induced autism model.

Reg.	Category	Sub-category	Entrez gene ID	Gene symbol	Fold Change
Down-regulation	GTs	CSGlcA-T	316533	*Chpf*	–1.21
		CSGlcA-T	296733	*Chpf2*	–1.5
		Fucosyl-T	84597	*Fut9*	–5.51
		GalNAc-T	304571	*Galnt9*	–1.51
		GalNAc-T	288611	*Galnt17*	–1.37
		GalNAc-T	64828	*B4galnt1*	–1.24
		GalNAc-T	117108	*B3gat1*	–1.19
		GalNAc-T	309105	*B4galnt4*	–1.16
		Gal-T	362275	*B4galt5*	–1.22
		GlcNAc-T	29582	*Mgat3*	–1.19
		GlcNAc-T	65271	*Mgat5*	–1.54
		GlcNAc-T	56819	*Extl3*	–1.44
		GlcNAc-T	361368	*Large1*	–1.29
		Glc-T	171129	*Uggt1*	–1.45
		Man-T	362465	*Tmtc1*	–1.40
		Man-T	308519	*Dpy19l3*	–1.40
		Sia-T	301155	*St6gal2*	–1.62
		Sia-T	364901	*St8sia5*	–1.36
		Sia-T	25547	*St8sia3*	–1.28
		Xyl-T	64133	*Xylt1*	–1.59
		**	245960	*Alg10*	–1.58
		**	290794	*Tnks*	–1.32
		**	363160	*Stt3b*	–1.26
	GHs	Hyaluronidases	367166	*Hyal1*	–1.44
		Hyalurononglucosaminidase	308797	*Cemip*	–1.51
		Hyalurononglucosaminidase	309400	*Tmem2*	–1.64
		Lysozomal Enzymes	367562	*Gaa*	–1.19
		Lysozomal Enzymes	684536	*Gba*	–1.19
		Mannosidases	295319	*Man1a2*	–1.27
		Mannosidases	308757	*Man2a2*	–1.30
		Nucleotide Synthesis	114711	*Gne*	–1.76
Up-regulation	GTs	Ribosyltransferase	24465	*Hprt1*	1.20
		Ribosyltransferase	117544	*Ppat*	1.35
		Sia-T	363040	*St3gal4*	1.28
		**	290029	*Pnp*	1.36
	GHs	Fucosidase	292485	*Fuca2*	1.25
		Nucleotide Synthesis	294673	*Hexb*	1.20
		**	25211	*Lyz2*	1.39

**Means sub-category is uncertain.

**TABLE 5:** The expression changes of glycan genes encoding glycoconjugates in valproic acid (VPA)-induced autism model.

Reg.	Category	Entrez gene ID	Gene symbol	Fold change	GO functions
Down-regulation	Lectins	50687	*L1cam*	–1.37	Axon guidance receptor activity
		64202	*Calr*	–1.20	Calcium ion binding
	GPI-anchored proteins	288280	*Ncam2*	–1.58	Axonal fasciculation
		24586	*Ncam1*	–1.26	Axonal fasciculation Brain development
		25356	*Cntn2*	–1.39	Axonogenesis
		58920	*Gpc1*	–1.24	Schwann cell differentiation
	Proteoglycans	25592	*Agrn*	–1.51	Chemical synaptic transmission
		56782	*Srgn*	–1.29	Apoptotic process
		54226	*App*	–1.18	Astrocyte activation
	GAG binding proteins	29715	*Slc8a1*	–1.82	Calcium ion export
		65047	*Slit1*	–1.71	Axon extension involved in axon guidance; Axon guidance
		83467	*Slit3*	–1.62	Axon guidance; brain development
		687064	*Col25a1*	–1.58	Axonogenesis in innervation; Extracellular matrix organization
		116683	*Efna5*	–1.51	Axon guidance; Brain development
		140447	*Slc8a2*	–1.29	Cellular calcium ion homeostasis Cognition and learning
		310207	*Sema5a*	–1.19	Axonal fasciculation; Axon extension
Up-regulation	Lectins	29403	*Asgr2*	1.91	Glycoprotein metabolic process
	GAG binding proteins	29366	*Serpine2*	1.27	Glycosaminoglycan binding
		25352	*Sod3*	1.26	Copper ion binding
		25728	*Apoe*	1.29	Aging

Among these 107 DEGGs, 27 genes were ≤ −1.5 fold down-regulated in the VPA-treated group, whereas eight genes were ≥ 1.5 fold up-regulated. The expression of these DEGGs with fold change of more than 1.5 fold was visualized as a heat map using MeV cluster software (Figure 4B). Notably, the color-based view demonstrated these DEGGs exhibiting the most significant fold change were clustered side-by-side in the dendrogram. To confirm the outcome of the RNA sequencing, we determined expression levels of 5 DEGGs (*Large1, Man2a2, St6gal2, Galnt9*, and *Hs3st5*) by qRT-PCR (Figure 4C). These genes were selected mainly because they have been reported as candidate genes related to autism. In VPA rats, the expression of 4 genes (*Large1, Man2a2, St6gal2*, and *Galnt9*) was significantly decreased, and the presentation of *Hs3st5* was greatly increased, which was consistent with results from RNA-seq.

### Integration of gene expression of anabolic enzymes and glycan patterns

**FIGURE 5:**
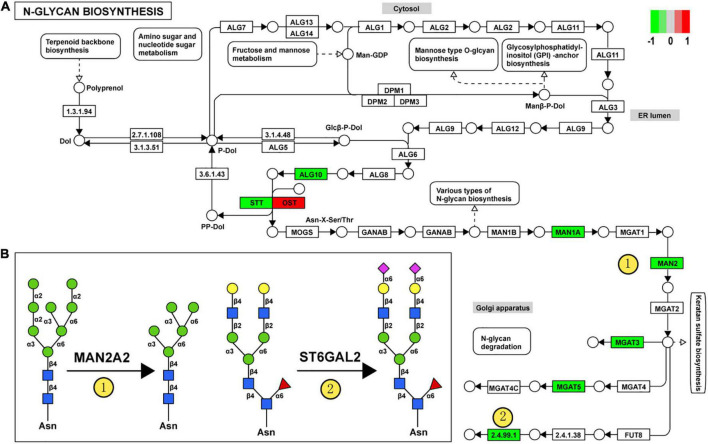
Relationship between glycan patterns and transcript levels of anabolic enzymes on the glycan biosynthetic pathway. **(A)** Differentially expressed glycan-related genes in N-glycan biosynthesis Kyoto encyclopedia of genes and genomes (KEGG) pathway. The transcription level of *Dad1, Alg10, Man1a2, Man2a2, Mgat3, St6gal2*, and *Mgat5* was decreased in the valproic acid (VPA)-induced autism model, which is highlighted with green. Only the *Stt3b* gene was increased by VPA treatment and was accentuated with red. **(B)** Schematic representation of the putative glycan changes under enzyme catalysis, including MAN2A2 and ST6GAL2. The symbol nomenclature for glycans was used to visualize the glycan structures.

For clarity, these DEGGs were divided into two categories: one is genes encoding enzymes involved in glycosylation, and the other is genes encoding glycoconjugates. Functional enrichment analysis revealed that these 51 DEGGs encoding anabolic enzymes primarily affected the N-glycan biosynthesis pathway (KEGG: rno00510). As shown in Figure 5A, members of the process of trimming to form the core structure and glycan extension from core structure were significantly downregulated in VPA rats, including *Man1a2, Man2a2, Mgat3, Mgat5*, and *St6gal2*. These results suggest that the synthesis of N-glycans might be inhibited in VPA-treated rats, which may be responsible for the alteration in glycan patterns with VPA treatment.

Furthermore, when combined with the results of lectin microarrays and the transcripts of glycan-related genes simultaneously, we found that alteration in transcript levels of anabolic enzymes in the N-glycan biosynthesis pathway is associated with corresponding changes in glycan structures of glycoproteins in VPA rats. Figure 5B showed the specific activity of MAN2A2 and ST6GAL2 enzymes in the N-glycan biosynthesis. *Man1a2* and *Man2a2* encode α-mannosidases, which could catalyze the trimming of high-mannose glycan and remove the mannose residue of glycan. In VPA-treated rats, α-mannosidases expression was significantly reduced at the mRNA level (*Man1a2*, FC = −1.27; *Man2a2*, FC = −1.30) (Figure 4C; Table 4), with consequent increased branched high-mannose glycan structures (recognized by ConA lectin). Our lectin microarrays also showed that a decrease in sialylated Gal/GalNAc (recognized by WGA and MAL-I lectin) corresponded to the down-regulated expression of the *St6gal2* gene (FC = −1.62) (Table 4). Sialyltransferase ST6GAL2 transfers Sia from CMP-sialic acid to Galβ1/GalNAc structure on glycoproteins. Taken together, the results of our study revealed a relationship between the mRNA levels of anabolic enzymes and the forms of glycans on glycoproteins during prenatal exposure to VPA.

### Functional sub-network of differentially expressed genes encoding glycoconjugates

**FIGURE 6:**
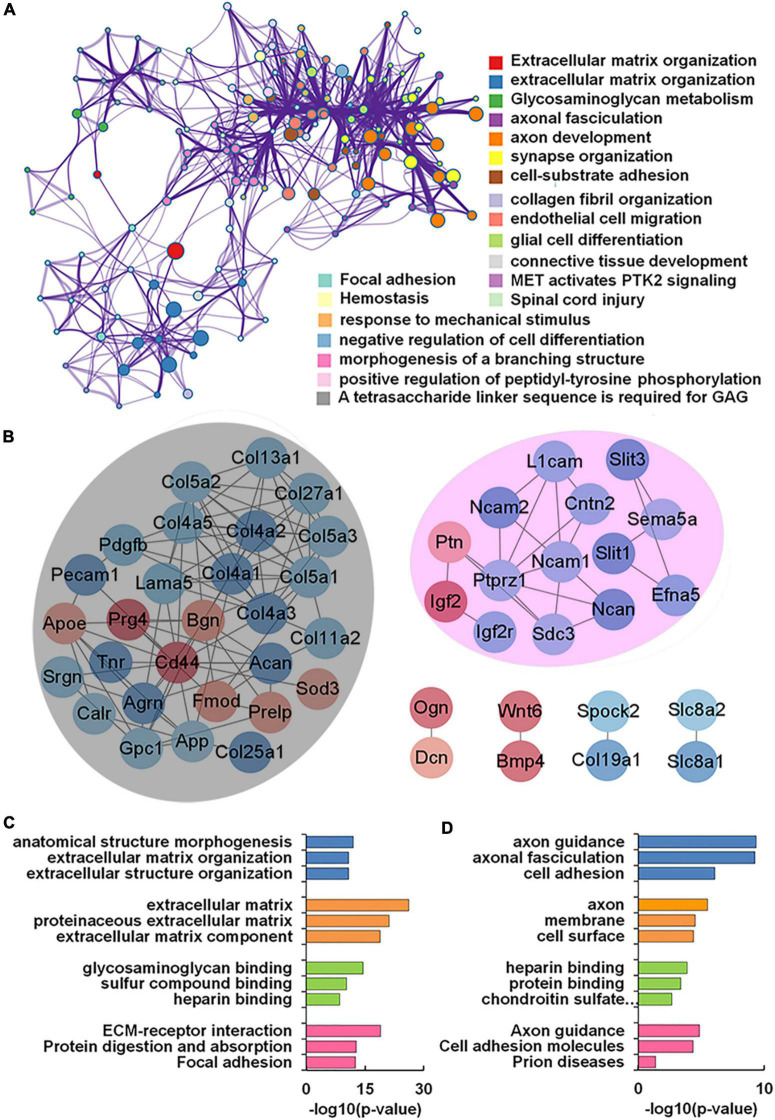
Functional network analysis of differentially expressed glycan-related genes (DEGGs) encoding glycoconjugates in valproic acid (VPA)-induced autism model. **(A)** The enriched network of representative GO terms of 56 DEGGs encoding glycoconjugates using Metascape. **(B)** Protein-protein interaction network of 56 DEGGs encoding glycoconjugates using Cytoscape software. Red and blue nodes represented up-and down-regulated DEGGs, and the color intensity was proportional to the expression. **(C,D)** Functional classification of differentially expressed genes of each cluster using Cytoscape plugin BiNGO based on universal GO and KEGG annotation terms.

Our transcriptomics results also showed 56 genes encoding glycoconjugates with statistical differences (*p* < 0.05) in VPA-treated rats. GO and KEGG enrichment analysis revealed that these 56 genes were annotated as extracellular matrix organization, axon development, and synapse organization (Figure 6A). We further constructed the protein-protein interaction network of differentially expressed glycoconjugates in an attempt to come out with molecular mechanisms in VPA rats. The relationships between the differentially expressed glycoconjugates were revealed from the STRING database, and MCL clustering was performed on the network using a confidence cutoff of 0.4. We noticed that MCL clustering formed two clusters (Figure 6B), and utilized the Cytoscape plugin BiNGO to perform biological process enrichment analysis with the differentially expressed genes of each set (Figures 6C,D). Notably, differentially expressed glycoconjugates in the black set were associated with extracellular matrix organization (GO: 0030198) and extracellular matrix (GO: 0031012). The most significantly enriched gene in the KEGG pathway out of these genes was ECM-receptor interaction (rno04512). In addition, glycoconjugates in the pink set were mainly involved in axon guidance (GO: 0007411), axonal fasciculation (GO: 0007413), and negative chemotaxis (GO: 0050919). Concerning molecular functions, target glycoconjugates were enriched in protein binding (GO: 0005515) especially, heparin-binding (GO: 0008201). The most significantly enhanced KEGG pathway was Axon guidance (rno04360). Overall, the functional effect of glycoconjugates was mainly correlated to extracellular matrix and axon guidance, which provide insights into the underlying molecular mechanism by which synaptic protein may undergo aberrant glycosylation during prenatal VPA exposure.

## Discussion

Here we show for the first time that there are aberrant expressions of glycan patterns and glycan-related genes in an environmentally triggered autism model in rats. Our lectin microarrays showed that high-mannose and Siaα2-3 Gal/GalNAc structure altered significantly in rats prenatally exposed to VPA. Using RNA-seq technology, 107 glycan-related genes were identified as differentially expressed genes in VPA rats. Some of these genes have been reported as candidate genes related to autism, including *Large1, Galnt9*, and *Hs3st5*. A functional study of DEGGs encoding anabolic enzymes found that trimming to form the core structure and glycan extension from core structure was primarily affected, which is consistent with alterations in glycan patterns. In addition, functional effect of DEGGs encoding glycoconjugates was mainly correlated to extracellular matrix and axon guidance. These results provide molecular evidence for the altered expression of genes responsible for the regulation of glycosylation in a VPA-induced rat model of autism.

A significant finding in the present study was that there was 14 lectins expression changes in VPA rats, and some of these alterations in glycan patterns were consistent with the previous publications. Our lectin microarrays showed that ConA was the up-regulation lectin with the most significant fold changes (FC = 1.82; Table 3) in VPA-treated rats, which binds to branched high-mannose Manα1-3Man. Another study also confirmed that prenatal exposure to VPA significantly increased high mannose-type N-glycan patterns in the hippocampal region, which is recognized by tomato lectin and serves as a biomarker for microglial cells (Codagnone et al., 2015). Our results also showed that the expression of terminal GalNAc structure was down-regulated in VPA rats, which is recognized by BPL (FC = −2.06) and VVA (FC = −2.47) (Figure 3C; Table 3). GalNAc structure is present in the perineuronal net surrounding basket interneurons. Ariza et al. (2018) found the number of basket interneurons slightly decreased in the prefrontal cortex in autism patients, suggesting that GalNAc structure is down-regulated in the brain tissue of ASD patients. Using lectin microarray analysis, Qin et al. (2017) showed that the expression of Siaα2-3 Gal/GalNAc (recognized by MAL-I and MAL-II) was significantly changed in serum samples from ASD. Interestingly, the Siaα2-3 Gal/GalNAc structure (recognized by MAL-I and WGA) in our lectin microarray analysis was significantly down-regulated in the VPA rats. Both of these results suggest that the Siaα2-3 Gal/GalNAc structure is involved in the molecular mechanism of ASD.

To examine the global regulation of glycan patterns in the environmentally triggered autism model, we systemically investigated the expression profiles of glycan-related genes in a VPA-induced rat model of autism. Three genes that encode anabolic enzymes, which are among the 107 DEGGs, have been reported as candidate genes for autism, and they are *Large1, Galnt9*, and *Hs3st5* (van der Zwaag et al., 2009). *Large1* gene encodes Large dual-function glycosyltransferase, and the only known protein modified by Large is dystroglycan. Recent works have reported that the length of the Large-glycan can be altered by changes in *Large1* expression, which affects the ligand-binding capacity of α-dystroglycan (van der Zwaag et al., 2009; Dwyer and Esko, 2016). It has also been reported that mutations in the *Large1* gene induce abnormal glycosylation of α-dystroglycan and result in congenital muscular dystrophy (Goddeeris et al., 2013). Our expression profiles analysis showed that the *Large1* gene was down-regulated in VPA rats (Table 4), indicating that VPA-induced autism is closely related to the abnormal expression of autism candidate genes.

When we combined lectin microarrays and transcriptomics analysis, we found that alterations in gene expression of anabolic enzymes were consistent with changes in glycan patterns in VPA rats. The gene expression changes that encode anabolic enzymes in animal models are also compatible with the lack of function in ASD patients, which may facilitate the exploration of the association between glycosylation and VPA treatment. As forementioned, our findings demonstrated that ST6GAL2 and its responsible sialylated Gal/GalNAc structures were down-regulated in VPA rats. In addition to ST6GAL2, the down-regulated sialyltransferase genes also included *St8sia5* and *St8sia3* (Table 4). Recently, copy number loss and SNPs in ST8SIA2 have been reported in ASD patients (Demirci et al., 2019). Since ST8SIA5 and ST8SIA3 transfer Sia from CMP-sialic acid to NeuAcα2/3R or NeuAcα2/8R structure on glycolipids, it was found that the Sia level of the ASD group was lower than that of the control group (Yang et al., 2018; Demirci et al., 2019). The evidence suggests that the Sia signal pathway may be associated with ASD.

VPA treatment also caused significant changes in the levels of 56 genes encoding glycoconjugates in our glycan-related genes list. Functional enrichment analysis revealed that these 56 DEGGs were annotated as extracellular matrix organization, axon development, and synapse organization (Figure 6A). A previous study has confirmed that several genes related to glycoconjugates were markedly disturbed in the animal models of autism. It was reported that the expression of three genes encoding collagens was increased in the mPFC of rats prenatally exposed to VPA (Olde Loohuis et al., 2017). Gasparini et al. (2020) investigated the expression profile of the circular RNAs in the hippocampus of the BTBR T+tf/J mouse model of autism. They found that biological and molecular pathways of hippocampal DEGs were associated with heparan sulfate pathway. Both collagens and heparan sulfate proteoglycan are significant components of the ECM, which is tightly connected to the perineuronal nets (Mansour et al., 2019). Therefore, disturbances in the expression of ECM components could lead to altered signaling, disturbing proper cellular functioning, and, in the case of neurons, abnormal outgrowth and synaptic functioning (Irie et al., 2012; Mutalik and Gupton, 2021). In the VPA animal model, neurons of the mPFC displayed cytoarchitectural alterations (Mansour et al., 2019), excitatory/inhibitory imbalance (Santos-Terra et al., 2022) and altered synaptic plasticity (Rinaldi et al., 2008). Taken together, we found the aberrant expression of glycan patterns and glycan-related genes in the prenatal VPA exposure model of autism. The results provide insights into the underlying cellular mechanism by which synaptic protein may undergo aberrant glycosylation during ASD.

### Limitations

However, it should be noted that our study has several limitations. One limitation is that we didn’t investigate these alterations of glycan patterns and gene expression in other animal models of ASD (i.e., genetic models). Then, we did not use immunohistochemical staining to identify specific glycan changes. Lectin histochemistry is non-specific because one lectin can usually recognize multiple types of glycan structures, making it difficult to distinguish the subtle differences in glycan structures. Lack of protein confirmation for the targeted genes and of biochemical determination of the proposed glycan alterations weakens this study. However, the correlation between gene expression of anabolic enzymes and glycan patterns reported by our research are reflected in VPA-induced rat model of autism, which is novel in exploring the molecular mechanism of ASD and need to be further verified in a future research.

## Data availability statement

The datasets presented in this study can be found in online repositories. The names of the repository/repositories and accession number(s) can be found in the article/Supplementary material.

## Ethics statement

The animal study was reviewed and approved by Animal Care and Use Committee of Shaanxi Normal University.

## Author contributions

YL: conceptualization, data curation, and writing—original draft. YD and QZ: methodology and data curation. ZQ: investigation and writing—review and editing. JF: visualization and resources. WR: investigation and supervision. ZW: data curation, conceptualization, and writing—review and editing. YT: conceptualization and writing—review and editing. All authors have read and agreed to the published version of the manuscript.

## Footnotes

1
https://www.ncbi.nlm.nih.gov/sra

2
https://www.genome.jp/kegg/

3
http://www.cazy.org/

4
www.ncbi.nlm.nih.gov

5
http://metascape.org/gp/

6
https://pathview.uncc.edu/

7
https://cn.string-db.org/

8
http://www.genome.jp/kegg/glycan/

## Supplementary material

Supplementary Table 1 Information and regulation about 107 differentially expressed glycan-related genes (DEGGs) in valproic acid (VPA)-treated rats.
